# Therapeutic response and side effects of repeated radioligand therapy with ^177^Lu-PSMA-DKFZ-617 of castrate-resistant metastatic prostate cancer

**DOI:** 10.18632/oncotarget.7245

**Published:** 2016-02-08

**Authors:** Hojjat Ahmadzadehfar, Elisabeth Eppard, Stefan Kürpig, Rolf Fimmers, Anna Yordanova, Carl Diedrich Schlenkhoff, Florian Gärtner, Sebastian Rogenhofer, Markus Essler

**Affiliations:** ^1^ Department of Nuclear Medicine, University Hospital Bonn, Bonn, Germany; ^2^ Institute for Medical Biometry, Informatics and Epidemiology, University of Bonn, Bonn, Germany; ^3^ Department of Urology, University Hospital Bonn, Bonn, Germany

**Keywords:** PSMA, ^177^Lu, prostate cancer, radioligand therapy, PSA

## Abstract

Prostate-specific membrane antigen (PSMA) is highly expressed on prostate epithelial cells and strongly up-regulated in prostate cancer (PC), making it an optimal target for the treatment of metastasized PC. Radioligand therapy (RLT) with ^177^Lu-PSMA-DKFZ-617 (Lu-PSMA) is a targeted therapy for metastatic PC. In this study, we retrospectively analyzed the side effects and the response rate of 24 hormone and/or chemorefractory PC patients with a mean age of 75.2 years (range: 64–82) with distant metastases and progressive disease according to the PSA level, who were treated with Lu-PSMA. Median PSA was 522 ng/ml (range: 17–2360). Forty-six cycles of Lu-PSMA were performed. Of the 24 patients, 22 received two cycles. Eight weeks after the first cycle of Lu-PSMA therapy 79.1% experienced a decline in PSA level. Eight weeks after the second cycle of Lu-PSMA therapy 68.2% experienced a decline in PSA relative to the baseline value. Apart from two cases of grade 3 anemia, there was no relevant hemato- or nephrotoxicity (grade 3 or 4). These results confirmed that Lu-PSMA is a safe treatment option for metastatic PC patients and has a low toxicity profile. A positive response to therapy in terms of decline in PSA occurs in about 70% of patients.

## INTRODUCTION

Prostate cancer (PC) is the second most common cancer in men worldwide and the fourth most common cancer overall [1]. Although the five-year survival rate in patients with localized PC is around 100%, this rate drops to 31% in patients with distant metastases [2]. Progression to androgen independence is the main cause of death in PC patients [3]. Most deaths related to PC are due to advanced disease. Prostate-specific membrane antigen (PSMA) is highly expressed on prostate epithelial cells and strongly upregulated in PC. The levels of PSMA expression are directly correlated with androgen independence, metastasis, and PC progression [4]; therefore, PSMA is an attractive target for the diagnosis and therapy of metastasized PC. ^177^Lu-PSMA-DKFZ-617 (Lu-PSMA), which is a DOTA derivative of the Glu-urea-Lys motif, was developed for the treatment of patients with metastatic PC [5, 6]. We recently published our first experiences with Lu-PSMA, which indicated that this therapy is safe and has a low early side effect profile [7]. A relevant decline in PSA was detected in 70% of patients. In the current study, we retrospectively analyzed the side effects and response rate in 24 patients who received up to two cycles of therapy with Lu-PSMA.

## RESULTS

Forty-six cycles of Lu-PSMA (mean of 6.0 GBq (range 4.1 – 7.1 GBq)) were performed in 24 consecutive hormone and/or chemorefractory patients with a mean age of 75.2 years (range: 64–82). Twenty-two patients received two cycles of therapy. One patient with multiple liver metastases and ECOG 3 died 10 weeks after the first cycle. The majority of his liver metastases did not express any relevant PSMA in ^68^Ga-PSMA PET. One patient did not receive the second cycle because of an L4 fracture, diagnosed 6 weeks after the first cycle. This patient underwent a spinal fusion. Twenty-two patients had a history of or were under therapy with enzalutamide and/or abiraterone. Twelve patients had received ^223^Ra-dichloride (1–6 cycles; median 5 cycles) (Table 1). The extent of metastases is shown in Table 2. Eleven patients also had local recurrence. The mean and median PSA levels prior to therapy were 628.3 and 522 ng/ml, respectively (range: 17.1–2360 ng/ml).

**Table 1:** The prior therapies

Therapy	History of n(%)	ongoing n(%)^2^	No history of n(%)
Prostatectomy	13 (54.2 %)		11 (45.8 %)
Abiraterone*	13 (54.2 %)	5 (20.8 %)	6 (25 %)
Enzalutamide^1^	5 (20.8 %)	9 (37.5 %)	10 (41.7 %)
Chemotherapy	10 (44.4 %)		14 (58.3 %)
Bisphosphonate or RANKL^+^ inhibitor	3 (12.5%)	17 (70.8 %)	4 (14.8%)
Ra-223	12 (50 %)		12 (50 %)

1 Ten patients received both abiraterone and enzalutamide. All these patients had received abiraterone prior to enzalutamide.

2 The hormone therapies with abiraterone or enzalutamide was not discontinued despite refractory situation according to the continuing PSA elevation under these medications

* Two patients had neither abiretarone nor enzalutamide, 1 patient took bicalutamide

+ Receptor activator of nuclear factor kappa-B ligand

**Table 2:** Extent of the disease in 24 patients, detected by PSMA-PET/CT

	Number of the patients (%)	Extent
Local recurrence	11 (45.8 %)	
Bone metastases	24(100 %)	< 6 metastases in 2 patients (8.3%) > 20 metastases in 22 patients (91.7%)
Lymph node metastases	20 (83.3 %)	ilical and abdominal in 8 patients (40%)thoracal in 1 patient (5 %) ilical to thoracal in 11 patients (55 %)
Liver metastases	3 (12.5 %)	singular metastasis in 1 patient multiple metastases in 2 patients

Fifteen patients (62.5%) exhibited good Eastern Cooperative Oncology Group (ECOG) performance status scores (0 or 1). Eight patients exhibited an ECOG score of 2 and one an ECOG score of 3. The blood and renal parameters prior to the first cycle are shown in Table 3. Four patients had received blood transfusion prior to therapy because of grade 3 tumor anemia 14 – 180 days prior to PSMA therapy (median 19.5 days). The tumor parameters are listed in Table 4.

**Table 3:** The blood, renal and hepatic parameters prior the first cycle

Parameter	min	max	mean	median
**Blood parameters**				
WBC (G/l) (norm: 3,6-10,5)	3.3	12.2	6.4	6.3
Hg (g/dl) (norm: 12,5-17,2)	9.0*	14.5	10.9	10.9
PLT (G/l) (norm: 160-370)	62	557	258	252
**Renal parameters**				
Creatinine (mg/dl) (norm: 0,6-1,3)	0.53	1.45	0.9	0.9
GFR (ml/min) (norm: > 70)	50	>70^+^		
TER MAG3 (ml/min/1,73 m^2^ BSA)	92^§^	363	200	203
**Liver function tests**				
Bilirubin total (mg/dl) (norm: 0,2-1,0)	0.28	1.20	0.47	0.40
ALT (U/l) (norm: < 50)	8	25	16	15
AST(U/l) (norm: < 50)	10	90	30.6	22.5
GGT (U/l) (norm: < 55)	17	181	50	38.5
Albumin (g/l) (norm: 35-52)	25.8	45.5	37.9	38.2

* Four patients had received blood transfusion prior to therapy because of grade 3 tumor anemia 14 – 180 days prior to Lu-PSMA therapy (median 19.5 days). The Hg values are the values from the blood test 1 day before treatment

+ 19 patients (79.2 %) had a GFR > 70 ml/min

§ 3 patients had low TER MAG3

WBC: white blood cells; Hg: Hemoglobine; PLT: Platelets; GFR: glomerular fraction rate; TER: tubular extraction rate; ALT: alanine transaminase; AST: aspartate transaminase; GGT: gamma-glutamyl transferase

**Table 4:** Tumor parameters

Parameters	min	max	mean	median
ALP (U/l) (norm: 34-117)	56	1607	259.3	147.5
LDH (U/l) (norm: < 248)	147	1875	396.3	247
CRP(mg/l) (norm: < 3)	0.2	128	21.4	15.3
PSA (ng/ml)	17.1	2360	628.3	522

ALP: alkaline phosphatase; LDH: lactate dehydrogenase; CRP: C-reactive protein: PSA: Prostate-specific antigen

### Response evaluation 2 months after the first cycle

**Figure 1:**
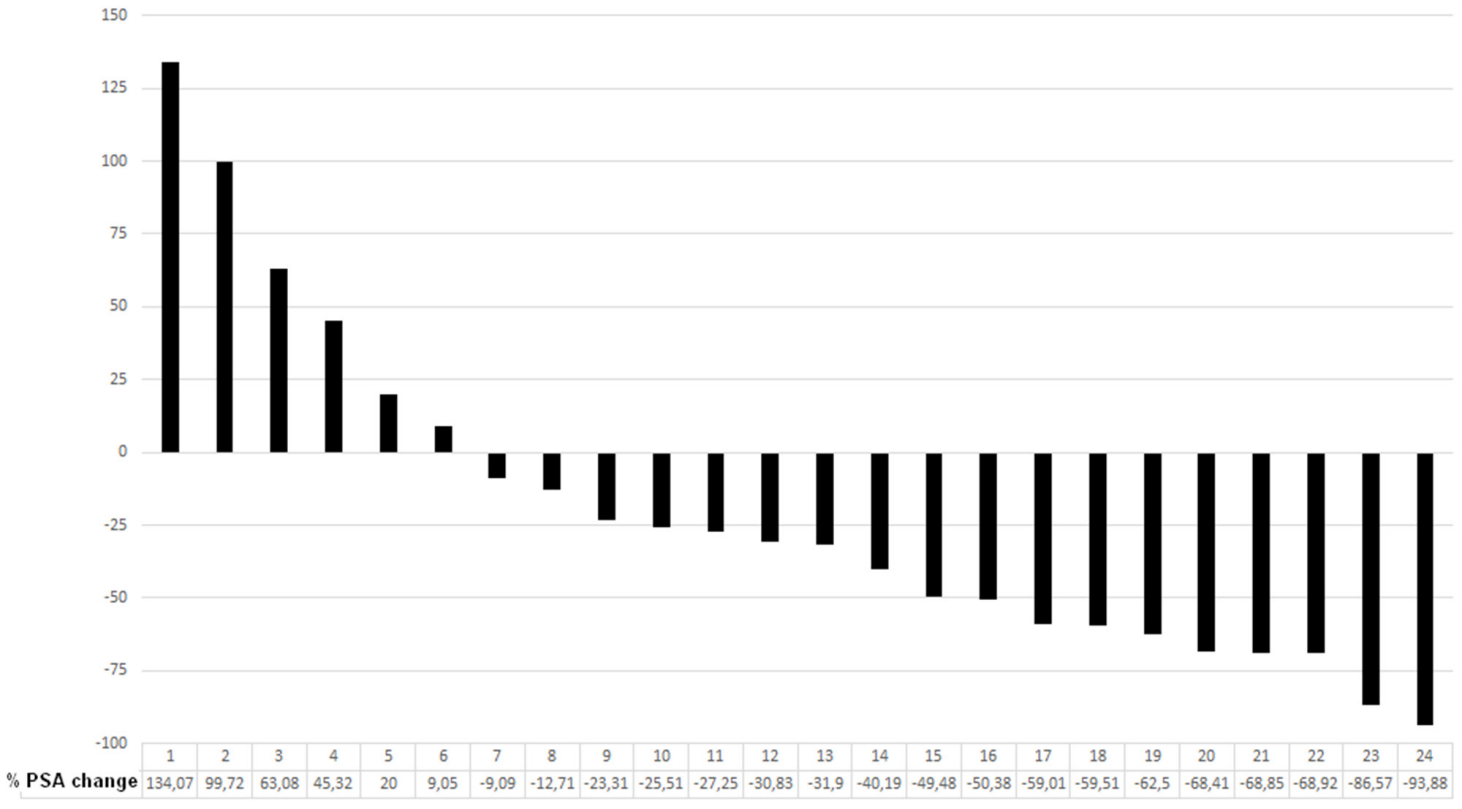
Waterfall plot showing percentage PSA change from baseline at 8 weeks after the first cycle in 23 patients (one patient with multiple liver metastasis died 10 weeks after the first cycle) 79.1 % of patients showed any PSA decline. 41.6 % of patients showed more than 50 % PSA decline.

Eight weeks after the first cycle of Lu-PSMA therapy 19/24 patients (79.1%) experienced a PSA decline, out of whom 13 experienced a decline of more than 30% and 10 more than 50% (41.6%). Five patients showed progressive disease according to the increase in PSA (Figure 1).

### Response evaluation 2 months after the second cycle according to PSA changes and PSMA PET/CT scan

**Figure 2:**
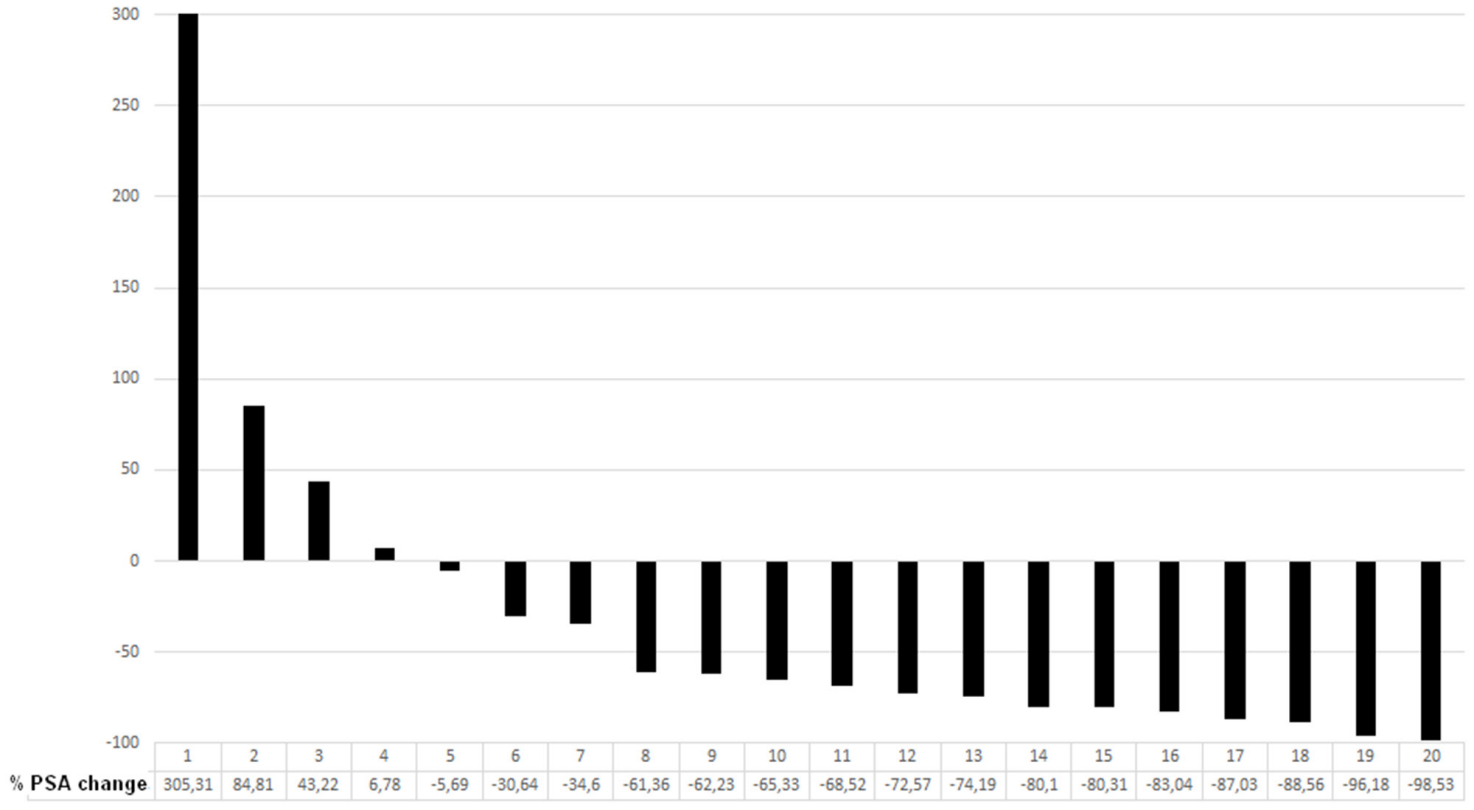
Waterfall plot showing percentage PSA change from baseline at 8 weeks after the second cycle in 19 patients /22 (3 patients died within 10 weeks after the second cycle).

**Figure 3:**
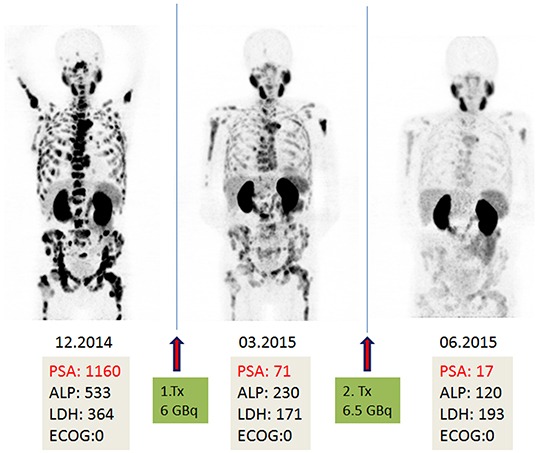
A 75-year-old patient with diffuse bone and lymph node metastases as well as local recurrence (left MIP image). History of chemotherapy and therapy with abiretarone, PSA elevation under enzalutamide. The patient underwent PSMA therapy as the last possible option. Continuing PSA decline and partial response in Ga-PSMA PET images after the first (middle MIP image) and second cycles (right MIP image).

Twenty-two patients underwent two cycles of Lu-PSMA therapy. Three of them died within 10 weeks after the second cycle; the PSA value of two of these was not measured. Eight weeks after the second cycle of Lu-PSMA therapy 15/22 patients (68.2%) experienced a PSA decline in comparison to the baseline PSA value, of whom 15 experienced a decline of more than 30% and 13 (60%) of more than 50%. Seven patients showed progressive disease according to the increase in PSA or disease progression (Figure 2). Twenty patients received PSAM PET/CT 6–8 weeks after the second cycle. PSMA PET showed a significant correlation to PSA changes (p=0.004) (Table 5 & Figure 3). Although the CT showed a significant correlation to PSA changes, the evaluation of the response by using PSMA-PET was more precise than the CT (Table 5). Of the three patients with liver metastases, one with PSMA negative liver metastases died 10 weeks after therapy, one with singular PSMA positive liver metastasis showed a stable disease in the liver and one with multiple PSMA positive liver metastases showed a partial response in the liver as well as in the extrahepatic manifestations.

**Table 5:** Response evaluation using PSMA PET/CT in 20 patients after the second cycle of Lu-PSMA therapy

	PSMA PET			CT			total
	PR	SD	PD	PR	SD	PD	
PSA decline	15	0	0	7	8	0	15
PSA elevation	1	0	4	0	4	1	5
total	16	0	4	8	11	1	20
p-value		0.004			0.04		

CT: computed tomography; PET: positron emission tomography; PD: progressive disease; PR: partial response; PSA: prostatic specific antigen; SD: stable disease.

### Predictors of a negative response

In this small number of patients we evaluated different parameters that may affect the PSA response, including prior therapies, age, ECOG, Gleason-score, baseline CBC, renal function and PSA level, as well as the need for pain medications. The only significant factor that negatively affected the PSA response was the regular need for opioid pain medications (0.004).

### Complaints and side effects in the first 2 days after administration, 4 weeks and between 4–8 weeks

No patient experienced any side effects immediately after injection of Lu-PSMA. No significant change in blood pressure or body temperature was observed. Table 6 shows the complaints and subjective side effects in relation to the number of patients as well as therapies. The most common side effect in the first 48 hours after injection was mild nausea (in 12.5% of patients) with no more than one time vomiting maximally. Nausea was easily controlled with ondansetron. Otherwise, the patients tolerated therapy very well. Fatigue was the most common complaint in patients after therapy, especially in the first 4 weeks. Dry mouth was reported in just 8.7% of cases and was transient and tolerable (Table 6).

**Table 6:** Complaints and side effects in the first 2 days, during the first 4 weeks and between 4- 8 weeks after administration

Complaints	48 p.i.	during the first 4 weeks after first and second cycle	4. – 8. weeks after first and second cycle
fatigue		8/46 Tx (17,4%)	6/46 Tx (13 %)
nausea and vomiting	4/46 Tx (8,7 %) 3/24 Pt (12,5 %)	2/46 Tx (4,3%)	4 /46 Tx (8,7 %)
dry lips/mouth	2/46 Tx (4,3%) 1/24 Pt (4,2 %)	3/46 Tx (6,5 %) 2/24 Pt (8,3 %)	4/46 Tx (8,7 %)
light headache	1/46 Tx (2,2%)		
hypogeusia		2/24 Pt (8.3 %)	2/46 Tx (4,3 %)
bone pain	2/46 Tx (4,3%) 1/24 Pt (4,2 %)	2/46 Tx (4,3 %) 1/24 Pt (4,2 %)	0

Tx: therapies; Pt: patients

Four patients needed 2 units of packed red blood cells (pRBC) prior to or after the second cycle, out of whom two patients received blood transfusion for the first time (see Figure 3). Apart from those patients who died after treatment, no patient experienced a relevant negative change in performance status.

### Hematotoxicity

**Figure 4:**
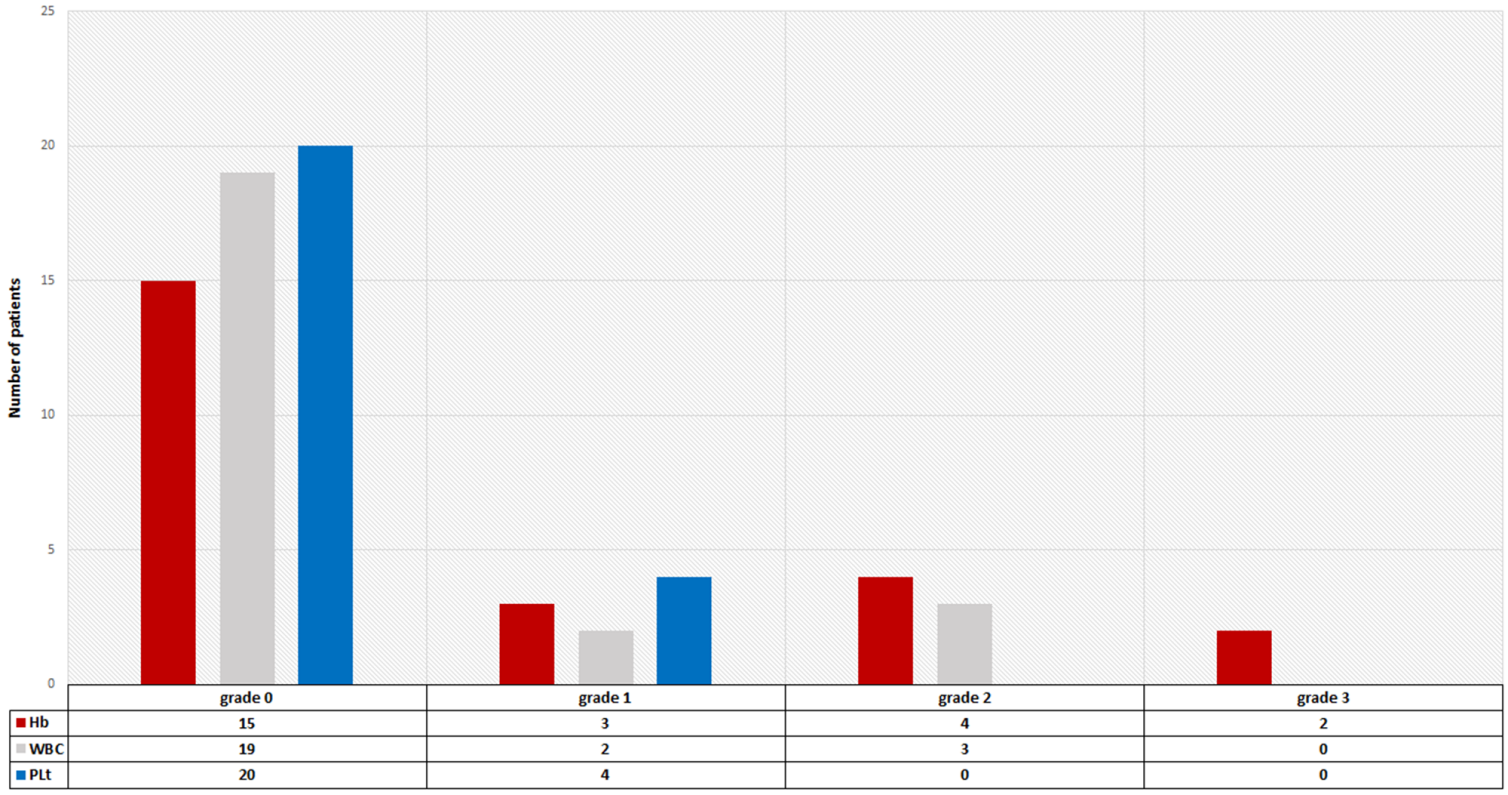
Hematotoxicity 2 months after the last cycle according to CTC criteria

The blood parameters prior to the first cycle of therapy are listed in Table 3. Relevant hematotoxicity (grade 3) occurred during the observation period (within 2 months after the last cycle) in just two patients. Apart from some grade 1 or 2 hematotoxicity the majority of patients did not show any hematotoxicity during the observation period (Figure 4).

### Nephrotoxicity and hepatotoxicity

**Figure 5:**
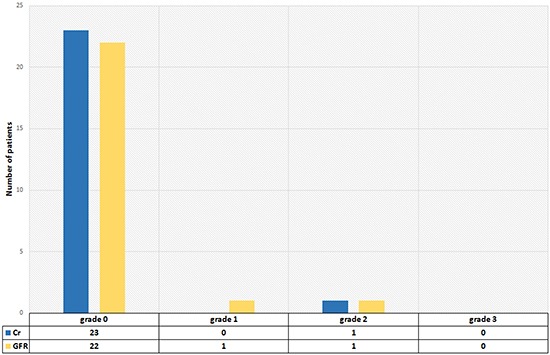
Nephrotoxocity 2 months after the last cycle according to CTC criteria

**Figure 6:**
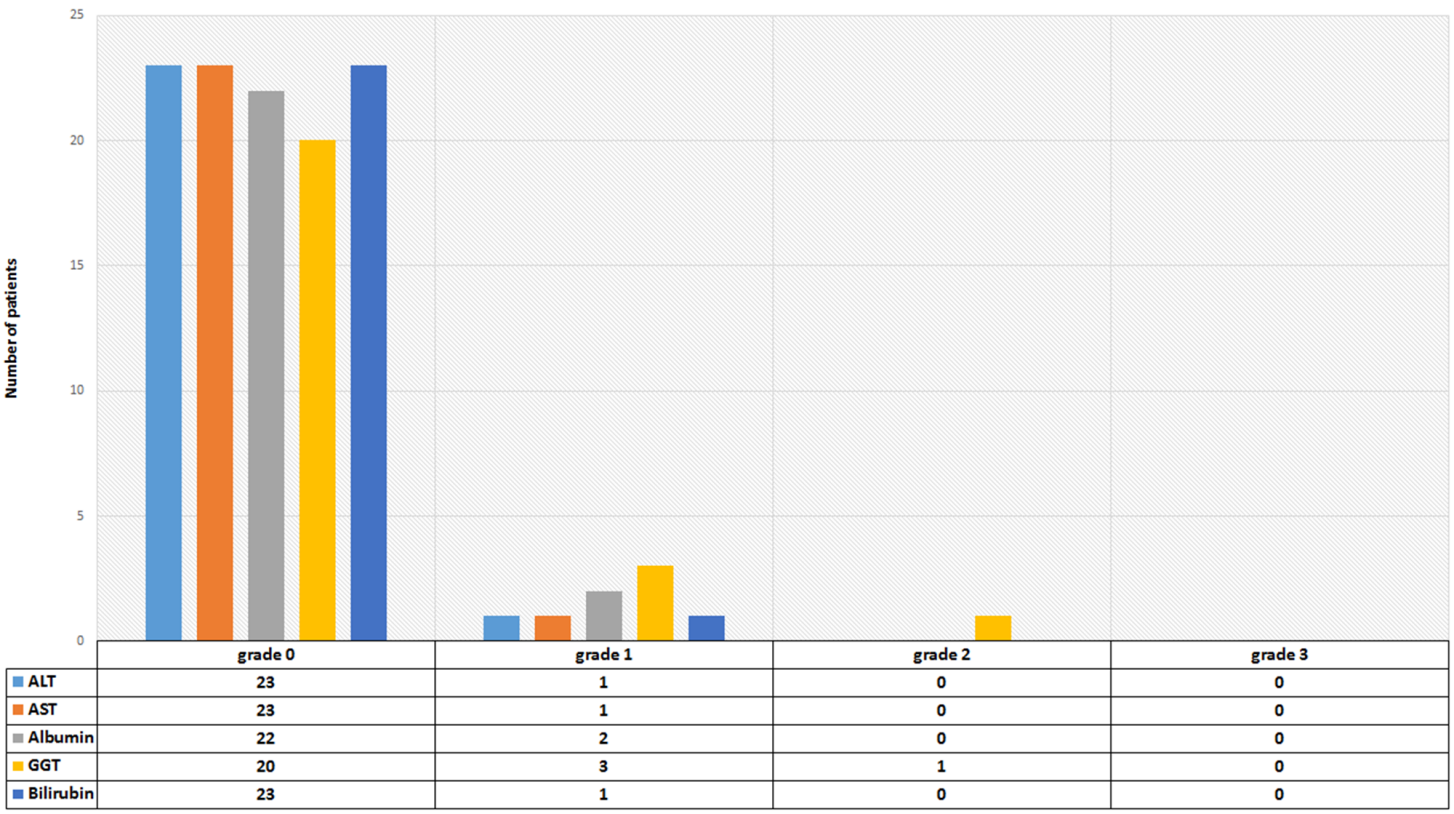
Hepatotoxocity 2 months after the last cycle according to CTC criteria

There was no relevant nephrotoxicity or hepatotoxicity (grade 3 or 4) (Figures 5 & 6).

## DISCUSSION

**Figure 7:**
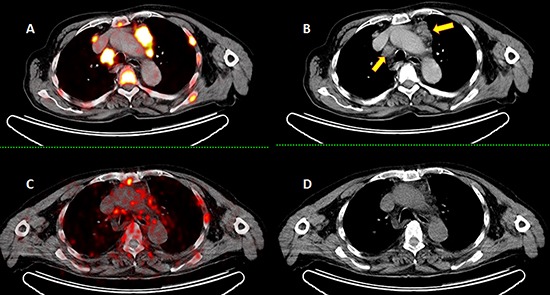
Ga-PSMA PET images **A&B.** of a 72-year-old patient with diffuse bone and lymph node metastases (yellow arrows show the enlarged lymph nodes) received two cycles of Lu-PSMA. Ga-PSMA PET images **C&D.** 2 months after the second cycle show a very good response with reduction in PSMA expression as well as a size reduction of the lymph node metastases in CT **B&D.**

Almost all patients with metastatic PC will initially respond to well-established and innovative anti-androgen treatments including the two recently approved hormone therapy agents, enzalutamide and abiraterone [10, 11], which significantly improve overall survival. Theranostics methods in Nuclear Medicine for the diagnosis and treatment of different cancer types have been developing rapidly, for example ^124^I/^131^I for thyroid cancer [12], ^68^Ga-DOTATATE/^177^Lu-DOTATATE for neuroendocrine tumor patients [13] and recently the ^68^Ga-PSMA/^177^Lu-PSMA for the treatment of PC [7, 14]. PSMA is an optimal target for the treatment of metastasized PC. Maresca et al. [15] described the design and synthesis of a series of small molecule inhibitors of PSMA. On the basis of this work, a preclinical evaluation of two radiopharmaceuticals, ^123^I-MIP-1072 and ^123^I-MIP-1095, which were designed to target PSMA in PC cells and tissue, was performed by Hiller et al. [6]. The study group from Heidelberg, Germany, showed the utility of ^131^I- MIP-1095 PSMA in the treatment of PC patients [16]. ^131^I has a half-life of 8.02 days with a high environmental radiation burden due to its high gamma energy, which limits its utility because of complex radiation protection regulations in most countries. In contrast to ^131^I, ^177^Lu gives a lower local dose rate, and therefore a lower radiation burden for staff and individual contacts. Furthermore the higher specific activity of ^177^Lu compared to commercially available ^131^I makes ^177^Lu preferable for targeted radionuclide therapies. In our recently published paper we showed the results and early side effects of Lu-PSMA therapy in a small cohort of ten patients after single dose of Lu-PSMA [7], which resulted in a PSA decline in seven patients, of whom five showed a decline of >50%. None of the patients experienced any side effects immediately after injection. Relevant hematotoxicity (grade 3 or 4) occurred in just one patient 7 weeks after radioisotope administration. Six patients did not show any hematotoxicity throughout the 8 weeks after therapy. There was no relevant nephrotoxicity or hepatotoxicity (grade 3 or 4). In the current paper we present a larger group of patients, who received up to two cycles of therapy. Our patients had no other therapy option and were selected very carefully for this treatment in cooperation with their urologists or oncologists. Similarly to our first study, 79.1% of patients experienced a PSA decline after the first cycle, out of whom 41.6% experienced a decline of more than 50%. Twenty-two patients underwent the second cycle, again with a PSA response in the majority of patients: 68.2% experienced a PSA decline, out of whom 60% experienced a decline of more than 50%. Twenty patients received a PSMA PET/CT 6–8 weeks after the second cycle, which showed a good correlation to the PSA changes; however, PSMA PET showed a better correlation (p=0.004) to PSA changes than CT alone (p=0.04). The reason for this difference is that despite a decline of PSMA expression the osteoblastic bone lesions did not show any change in size in this period of time. There were seven patients in whom a reduction in the size and number of lymph node metastases could be also detected in CT (Figure 7). These results are highly promising, because our patients had no other approved therapy option in routine practice. Fifty percent of our patients had also undergone Ra-223 therapy (median 5 cycles) as a therapy option approved by the FDA [17, 18]. The decision to administer Lu-PSMA after Ra-223 was based on the following criteria: 1) the presence of PSMA-positive metastases in Ga-PSMA PET after six cycles; 2) radiological progress under Ra-223 therapy; and 3) in two patients, a shortage of Ra-223 in November 2014 after one and four cycles of Ra-223, respectively. No prior therapies, including chemotherapy, hormone therapy and therapy with Ra-223, had either a negative or a positive effect on PSA response. The only factor that significantly correlated to PSA elevation was the regular need for opioid pain medications (p=0.004); however, this does not mean that these patients did not profit from this therapy. We need more patients with a longer follow-up to measure the overall survival in these patients.

In the current study as in the first study no patient experienced any side effects immediately after injection of Lu-PSMA. The most common side effect in the first 48 hours after injection was mild nausea (in 12.5% of patients) with a maximum of one episode of vomiting. Nausea was controlled easily with ondansetron in all cases. To date our patients have not received prophylactic antiemetics; however, this may be an option for patients who experience nausea during the first cycle.

One of our initial concerns about Lu-PSMA therapy was xerostomia, which could arise given the physiological uptake of this tracer by the salivary glands; however, the side effects up to 2 months after the second cycle were mild and transient and occurred in just 8.7% of patients; in all cases the symptoms were tolerable and patients only mentioned it after they were asked directly about this side effect.

Four patients had a history of blood transfusion(s) prior to the first cycle, out of whom one patient with ECOG 3 and multiple metastases died 10 weeks after the first cycle. Altogether five patients received blood transfusion prior to or after the second cycle, of whom two patients were transfused for the first time (grade 3 anemia). One limitation of every retrospective study without a control group is that differentiation between the therapy-induced side effects and the side effects due to progression of the disease is often difficult if not impossible, and therefore in our study every side effect, complaint and toxicity was considered to be therapy induced.

Tagawa et al. [19] treated 47 patients with Lu-PSMA mAb J591. In their study all patients experienced reversible hematological toxicity, with grade 4 thrombocytopenia occurring in 46.8% (29.8% received platelet transfusions) without significant hemorrhage. A total of 25.5% experienced grade 4 neutropenia. In the present study relevant hematotoxicity (grade 3) occurred during the observation period in just two patients. Apart from some grade 1 or 2 hematotoxicity the majority of patients did not show any hematotoxicity during the observation period (Table 3). This is probably due to the size of the mAb, which are large molecules and therefore show poor permeability in solid tumors and slow clearance from the circulation. This combination leads to suboptimal tumor targeting and an increase in the dose absorbed by the red marrow, narrowing the therapeutic window [16].

Due to the physiological expression of PSMA in the kidneys, there is concern regarding potential toxicity due to radiation to the kidneys. Zechmann et al. [16] reported that there was no apparent evidence or negative trend in either calculated GFR or serum creatinine levels in a one-year follow-up after therapy with ^131^I-MIP-1095 PSMA. In our study, no patient experienced grade 3 nephrotoxicity, and 22 patients (91.7%) did not experience any nephrotoxicity. We should mention here that after a 2-month follow-up we can rule out acute renal insufficiencies, but a longer follow-up is needed to determine any chronic side effects.

Kabasakal et al. [20] studied the absorbed dose of Lu-PSMA in different organs of seven patients with progressive PC who received a diagnostic dose of ^177^Lu-PSMA-617 with a mean activity of 192.6 ± 11.0 MBq. They showed that the highest estimated radiation doses were in the parotid glands and kidneys. Calculated radiation-absorbed doses per megabecquerel were 1.17 ± 0.31 mGy for the parotid glands and 0.88 ± 0.40 mGy for the kidneys. The radiation dose given to the bone marrow was significantly lower than that for the kidney and parotid glands (p < 0.05). Delker et al. [21] showed similar results. They acquired whole-body planar images and SPECT/CT images of the abdomen in five patients during two treatment cycles at approximately 1, 24, 48 and 72 h after administration of 3.6 GBq (range 3.4 to 3.9 GBq) Lu-PSMA. The calculated radiation-absorbed doses per megabecquerel were 1.4 mGy for the parotid glands and 0.6 mGy for the kidneys. According to these both studies [20, 21], although the kidneys are expected to be dose-limiting organs due to high radiotracer uptake, based on earlier experience obtained from conventional external beam radiotherapy, the maximum kidney dose is generally accepted to be 23 Gy, and in order to reach this dose limit cumulatively a mean of 30 GBq of ^177^Lu-PSMA can be given. However, these results should be proved in studies with more patients, who are being treated with about 6.0 GBq Lu-PSMA.

## MATERIALS AND METHODS

Twenty-four consecutive hormone and/or chemorefractory PC patients with distant metastases and progressive disease according to the PSA level were treated with Lu-PSMA between November 2014 and June 2015 in the Department of Nuclear Medicine (University Hospital Bonn). The prior therapies are listed in Table 1. All patients underwent a ^68^Ga-PSMA-11 (^68^Ga-PSMA) PET/CT prior to therapy to evaluate the PSMA expression status of the metastases. All patients had bone metastases and the majority of them (83.3%) had lymph node metastases. Local recurrence was detected in 11 patients (45.8%). Three patients also had liver metastases (Table 2). Written information regarding the therapy and its possible side effects was provided to each patient at two time points: first after the PET scan and once again 24 hours prior to therapy. The local ethics committee approved this retrospective study, and all subjects had provided prior written informed consent.

### Treatment planning

#### ^68^Ga-PSMA-11 PET/CT

^68^Ga-PSMA was applied via slow intravenous injection (30–60 sec) using a weight-adapted dose of 2 MBq/kg body weight in a total volume of 5–10 mL (diluted with 0.9% sterile sodium chloride solution), followed by 20 ml of sterile 0.9% sodium chloride. The average injected dose was 140 MBq. PET/CTs were performed on a Biograph 2 PET/CT scanner.

#### Renal function test and renal scintigraphy with ^99m^Tc-MAG3

Creatinine and glomerular function tests (GFR) were performed in all patients from prior to therapy to 2 months after the last cycle. To rule out any renal obstructive disease and to measure the tubular extraction rate of MAG3 (TER MAG3), all patients underwent renal perfusion scintigraphy with ^99m^Tc-MAG3 within 1 week prior to and 8 weeks after the last treatment. The scans were performed using dual-head SPECT cameras (AnyScan, Mediso). The procedure is described in detail elsewhere [8, 9].

#### Radioligand therapy (RLT)

PSMA was obtained from ABX GmbH (Radeberg, Germany). The preparation of Lu-PSMA was explained in detail in a previous publication [7].

The treatment solution was administered by slow intravenous injection over 1 minute followed by 1000 ml of NaCl or Ringer. In order to reduce therapy-induced damage to the salivary glands, the patients received ice packs over the parotid and submandibular glands from 30 min prior to and up to 4 hours after administration of the Lu-PSMA. All patients were discharged 48 hours after therapy according to the rules of the Federal Office for Radiation Protection in Germany (BfS).

#### Data collection and follow-up

One day prior to therapy, the hematological and renal status, liver function tests, tumor marker PSA, alkaline phosphatase, and blood biochemistry were evaluated in all patients. The ECOG performance-status score, therapy-induced side effects during the time of hospitalization and at follow-up, and laboratory examinations for at least up to 8 weeks after therapy were obtained in all patients. All patients were contacted by telephone regularly at 1- to 2-week intervals.

#### Tumor response evaluation

The tumor marker PSA was used as the main marker for the response evaluation. We classified the changes in PSA level as a decrease of more than 50%, more than 30%, and any decline. Any increase in PSA was considered as disease progression. Twenty patients received a second PSMA PET/CT scan 6–8 weeks after the second cycle of the Lu-PSMA therapy. The results of these scans were correlated with the PSA changes. The response on the CT images were evaluated according to the Response Evaluation Criteria in Solid Tumor (RECIST). The PSMA PET images were evaluated as follows: partial response: > 30% reduction of PSMA expression in the target lesions, progressive disease: > 30% increase of PSMA expression in the target lesions or developing new lesions. Stable disease: less than 30% change of PSMA expression. Up to five bone and lymph node metastases in each patient were selected as target lesions.

#### Toxicity

Toxicity was recorded using the Common Terminology Criteria for Adverse Events (CTCAE), version 4.0, and was analyzed according to the NCI guidelines for investigators.
